# Exercise Preconditioning Blunts Early Atrogenes Expression and Atrophy in Gastrocnemius Muscle of Hindlimb Unloaded Mice

**DOI:** 10.3390/ijms23010148

**Published:** 2021-12-23

**Authors:** Lorenza Brocca, Maira Rossi, Monica Canepari, Roberto Bottinelli, Maria Antonietta Pellegrino

**Affiliations:** 1 Department of Molecular Medicine, University of Pavia, 27100 Pavia, Italy; lorenza.brocca@unipv.it (L.B.); maira.rossi01@universitadipavia.it (M.R.); monica.canepari@unipv.it (M.C.); roberto.bottinelli@unipv.it (R.B.); 2 ICS-Maugeri (IRCCS), Scientific Institute of Pavia, 27100 Pavia, Italy; 3 Interdipartimental Centre of Biology and Sport Medicine, University of Pavia, 27100 Pavia, Italy

**Keywords:** disuse atrophy, hindlimb unloading, physical preconditioning, atrogenes

## Abstract

A large set of FoxOs-dependent genes play a primary role in controlling muscle mass during hindlimb unloading. Mitochondrial dysfunction can modulate such a process. We hypothesized that endurance exercise before disuse can protect against disuse-induced muscle atrophy by enhancing peroxisome proliferator-activated receptor-γ coactivator-1α (PGC1α) expression and preventing mitochondrial dysfunction and energy-sensing AMP-activated protein kinase (AMPK) activation. We studied cross sectional area (CSA) of muscle fibers of gastrocnemius muscle by histochemistry following 1, 3, 7, and 14 days of hindlimb unloading (HU). We used Western blotting and qRT-PCR to study mitochondrial dynamics and FoxOs-dependent atrogenes’ expression at 1 and 3 days after HU. Preconditioned animals were submitted to moderate treadmill exercise for 7 days before disuse. Exercise preconditioning protected the gastrocnemius from disuse atrophy until 7 days of HU. It blunted alterations in mitochondrial dynamics up to 3 days after HU and the expression of most atrogenes at 1 day after disuse. In preconditioned *mice*, the activation of atrogenes resumed 3 days after HU when mitochondrial dynamics, assessed by profusion and pro-fission markers (mitofusin 1, MFN1, mitofusin 2, MFN2, optic atrophy 1, OPA1, dynamin related protein 1, DRP1 and fission 1, FIS1), PGC1α levels, and AMPK activation were at a basal level. Therefore, the normalization of mitochondrial dynamics and function was not sufficient to prevent atrogenes activation just a few days after HU. The time course of sirtuin 1 (SIRT1) expression and content paralleled the time course of atrogenes’ expression. In conclusion, seven days of endurance exercise counteracted alterations of mitochondrial dynamics and the activation of atrogenes early into disuse. Despite the normalization of mitochondrial dynamics, the effect on atrogenes’ suppression died away within 3 days of HU. Interestingly, muscle protection lasted until 7 days of HU. A longer or more intense exercise preconditioning may prolong atrogenes suppression and muscle protection.

## 1. Introduction

Disuse-induced loss of muscle mass is a frequent phenomenon occurring in a variety of conditions such as immobilization following traumatic lesions, deconditioning, ageing, and in chronic diseases. Muscle atrophy is a major clinical problem. It exposes subjects to falls and fractures and favors further deconditioning. It is related to metabolic alterations, such as insulin resistance, as well as low-grade chronic inflammation [1], which are among the major risk factors of chronic diseases [2]. It worsens the prognosis of many chronic diseases [3], which benefit from exercise training [2,4].

A metabolic program, based on mitochondrial dysfunction and PGC1α down-regulation, is believed to play a major role in skeletal muscle atrophy. A deficit of the level of PGC1α, a master regulator of mitochondrial biogenesis [5,6,7,8], as well as failure in mitochondrial dynamics have detrimental consequences on muscle mass maintenance [9,10,11,12]. By using the hindlimb unloading (HU) model, we previously identified PGC1α and mitochondrial pro-fusion proteins’ deficiency as major causes of slow-twitch soleus and fast-twitch gastrocnemius disuse muscle atrophy, respectively [8,13]. The observation that muscle overexpression of PGC1α blunted or prevented HU-induced atrophy in both muscles by counteracting the activation of catabolic pathways strengthened the idea that mitochondrial dysfunction plays a major role in disuse atrophy [8,13].

Exercise is an attractive tool to prevent muscle wasting as it is known to cause mitochondrial adaptations and to enhance PCG1α expression. Indeed, PGC1α expression in skeletal muscle is highly inducible by physiological cues, including a single bout of exercise and endurance training in rodent models [14] and *human* subjects [15,16]. A recently published review [17] summarized the few but encouraging cases of evidence on the use of exercise as a tool to prevent disuse atrophy. More specifically, in *mice*, two weeks of concurrent exercise, a combination of endurance and sprint exercise, 7 days before HU, had a protective effect on gastrocnemius muscle mass [18]. In *rats*, contradictory results were obtained using a single bout of exercise before HU. Fujino et al. [19] showed blunting of soleus muscle atrophy after 2 weeks of HU, whereas Yoshihara et al. [20] showed no effect on gastrocnemius muscle atrophy following 1 week of HU.

FoxOs control a broad range of atrophy-related genes (atrogenes) required to cause disuse muscle atrophy, at least in *mice* [21,22]. The atrophy-related ubiquitin ligases, *MuRF1* and *Atrogin1* were the first genes to be identified as FoxO targets and to be used as markers of muscle atrophy. However, *MuRF1* and *Atrogin1* knockout *mice* were only partially or transiently protected from muscle loss after denervation, suggesting that other genes were also involved in protein breakdown [23,24]. Subsequently, new atrogenes were described [25]. We recently reported that 96% of genes related to HU- induced muscle atrophy are under FoxO control and seventeen are the core machinery for protein breakdown [26]. Interestingly, the gastrocnemius of *mice* lacking FoxOs is completely protected from HU atrophy, although *MuRF1* and *Atrogin1* are still increased. The suppression of other atrogenes, mainly involved in protein catabolism, proved fundamental in protecting muscle mass during disuse [26].

The mechanism of exercise-induced protection on muscle atrophy appears to be not yet clearly defined, and inconsistent evidence on several atrogenes is available in disuse following exercise preconditioning [18,19,20]. The prevention of some proteolytic markers’ up-regulation was found in the *rat* soleus [19], but no blunting effect on two major atrogenes, *MuRF1* and *Atrogin1*, was observed in *mice* gastrocnemii despite muscle protection [20], and variable results were reported in *rat* gastrocnemii [18].

Furthermore, FoxOs activation can be modulated by PCG1α [5] and by post-translational modifications, e.g., by the AMPK [27] and the SIRT1 deacetylase [28,29] that phosphorylates and deacetylates FoxO, respectively. Analyses of PGC1α adaptations with preconditioning have provided inconsistent results so far [18,20]. No information on AMPK and SIRT1 activation following preconditioning is available.

We hypothesize that exercise before disuse can protect against disuse-induced muscle atrophy by enhancing PGC1α expression and preventing mitochondrial fusion deficit and, in turn, atrogenes’ activation. The specific goals of this study were to assess (i) the impact of exercise preconditioning on mitochondrial dynamics and on a large set of FoxO-dependent atrogenes during disuse; (ii) the relationship between atrogenes’ expression and the known FoxO modulators AKT, PCG1α, AMPK, and SIRT1.

We focused our analysis on the Gastrocnemius (Gas) muscle as it goes through early atrophy, which is mainly due to the increase in catabolic processes triggered by a metabolic deficit, with a very limited impact of decreased protein synthesis [13]. Therefore, Gas appeared well suited to address the impact on disuse muscle atrophy of modulating mitochondrial dynamics by exercise preconditioning. We studied the full set of FoxO-dependent atrogenes we previously found to control muscle mass in the gastrocnemius following HU, as *Atrogin1* and *MuRF1* might not play a major role in the process [26]. Finally, we focused on short-duration hindlimb unloading since it is known that atrogenes’ expression is transient and occurs early in response to disuse [8,13,26].

## 2. Results

### 2.1. Physical Preconditioning, PGC1α and AMPK

**Figure 1:**
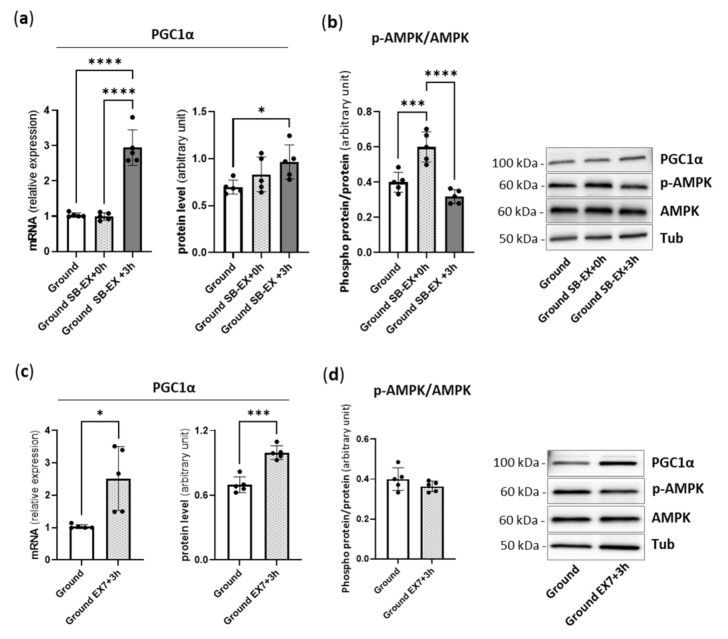
A single exercise bout and 7-day endurance training increase mRNA and protein expression of PGC 1α in ground *mice*. Gene expression and protein content analysis of PGC 1α (**a**) and activation level of AMP-kinase (**b**) in gastrocnemius of *mice* subjected to a single bout of treadmill exercise and relative representative Western blot; protein levels were normalized for tubulin expression. Ground: Control *mice*; Ground SB-EX + 0 h: *Mice* subjected to a single bout of exercise sacrificed immediately or 3 h after exercise (Ground SB-EX + 3 h). Gene expression and protein content analysis of PGC 1α (**c**) and activation level of AMP-kinase (**d**) in gastrocnemius of trained *mice* and relative representative Western blot; protein levels were normalized for tubulin expression. Ground: Control *mice*; Ground EX7 + 3 h: *Mice* subjected to 7 days of endurance exercise sacrificed 3 h after the last section of exercise. The activation level of AMP-kinase was determined through the ratio between the content of the phosphorylated (*p*) and total form. Data are presented as means ± SD * *p* < 0.05 *** *p* < 0.0005 **** *p* < 0.0001.

To examine whether the endurance exercise protocol was able to induce up-regulation of PGC1α, we investigated the expression and content of PGC1α in the gastrocnemius (Gas) muscle isolated from *mice* immediately after (ground SB-EX + 0 h) and 3 h after (ground SB-EX + 3 h) a single bout of treadmill running (Figure 1a), and 3 h after the last bout of treadmill running in 7 days in a daily endurance exercise program (ground EX7 + 3 h) (Figure 1c). PGC1α was significantly induced, both at mRNA and protein levels, compared to sedentary controls 3 h after, but not immediately after, a single bout of exercise (Figure 1a), and in ground EX7 + 3 h (Figure 1c). PGC1α content was ~25% higher following 7 days of endurance training than in ground Gas.

AMPK phosphorylation was higher, i.e., its activation was enhanced, immediately after a single bout of treadmill running (Figure 1b). It was not more phosphorylated than in ground *mice* 3 h after a single bout of treadmill running (Figure 1b) and 3 h after the last bout of treadmill running in the 7-day exercise program (Figure 1d).

### 2.2. Muscle Fibers Cross Sectional Areas (CSA)

**Figure 2:**
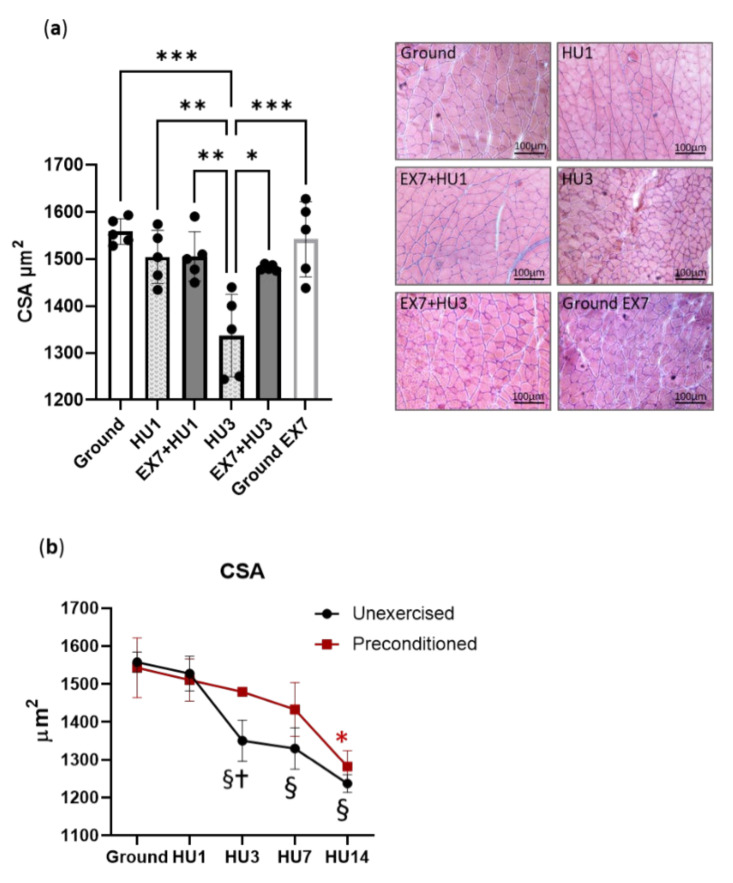
Physical preconditioning mitigates disuse-induced atrophy of gastrocnemius. Cross Sectional Area (CSA) of gastrocnemius muscle fibers of *mice* subjected to disuse alone and preceded by physical preconditioning and representative hematoxylin–eosin staining; scale bars: 100µm (**a**). CSA time course changes with disuse alone and preceded by 7 days of physical preconditioning (**b**). Ground: Control *mice*; HU1, HU3, HU7, and HU14: *Mice* subjected to 1, 3, 7, and 14 days of hindlimb unloading; Ground EX7: *Mice* subjected to 7 days of physical exercise. Data are presented as means ± SD * *p* < 0.05 ** *p* < 0.005 *** *p* < 0.0005. §: Different from ground and unexercised HU1 groups; †: Different from preconditioned HU3 group; ∗: Different from ground and preconditioned HU1 and HU3 groups.

To assess whether exercise before HU was able to protect muscle from atrophy, CSA of skeletal muscle fibers was determined on Gas cross-sections. No changes of muscle fibers’ CSA were found in Gas of *mice* both after 1 day of HU (HU1) and after HU1 preceded by 7 days of exercise (EX7 + HU1) (Figure 2a). Gas muscle underwent 13.3% muscle fiber atrophy after 3 days of HU (HU3). When HU3 was preceded by 7 days of exercise (EX7 + HU3), the muscle fibers’ CSA was significantly higher than HU3 and not different from ground *mice* CSA. To assess whether the impact of exercise could be due to the induction of muscle hypertrophy before HU, we measured the CSA of the Gas muscle of ground *mice* following the same exercise paradigm (ground EX7). No changes in fibers CSA were found.

To understand how long the protective effect of preconditioning on muscle mass lasted, we prolonged the disuse period to 14 days. In Figure 2b, mean values of Gas fibers’ CSA at 1, 3, 7, and 14 days of HU with and without exercise preconditioning are shown. Although the protective effect somewhat faded way, mean values of CSA of Gas muscle fibers from preconditioned *mice* were not lower than ground *mice* until 7 days into HU. At 14 days of HU, Gas fibers of preconditioned *mice* had the same size as those of *mice* subjected to disuse alone, i.e., the protective effect disappeared.

### 2.3. Atrophy-Related Genes

**Figure 3:**
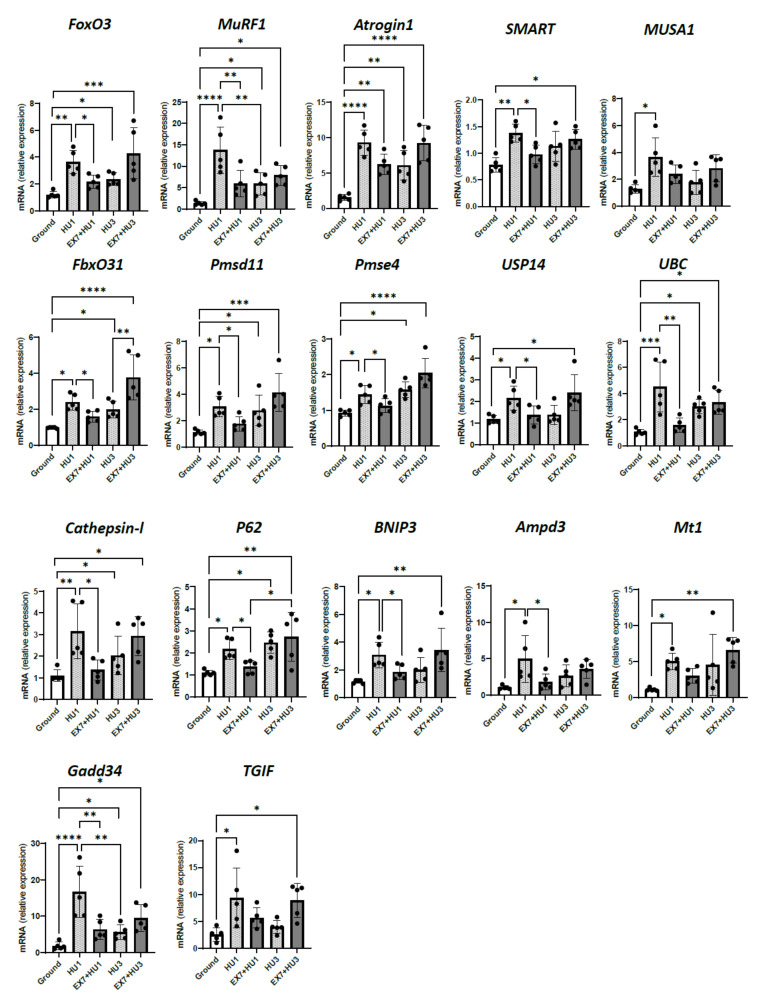
Physical preconditioning counteracts the early atrogenes’ induction during disuse. RT-PCR analysis of transcriptional levels of *FoxO3* and the muscle-specific ubiquitin ligases *MuRF1*, *Atrogin1*, *SMART*, *MUSA1*, *FbxO31*, proteasome subunit atrogenes (*Psmd11, Psme4*), de-ubiquitinating enzyme atrogene (*USP14*), ubiquitin atrogene (*UBC*), autophagy-related atrogenes (*Cathepsin l, P62, BNIP3*), AMP deaminase atrogene (*Ampd3*), oxidative stress related atrogene (*Mt1*), unfolded protein response atrogene (*Gadd34*), transcription regulators Smad2/3 atrogene (*TGIF*). The expression levels were normalized on *GAPDH*. Ground: Control *mice*; HU1: *Mice* subjected to 1 day of hindlimb unloading; EX7 + HU1: *Mice* subjected to 7 days of physical preconditioning followed by 1 day of hindlimb unloading; HU3: *Mice* subjected to 3 days of hindlimb unloading; EX7 + HU3: *Mice* subjected to 7 days of physical preconditioning followed by 3 days of hindlimb unloading. Data are presented as means ± SD * *p* < 0.05 ** *p* < 0.005 *** *p* < 0.0005 **** *p* < 0.0001.

To determine whether exercise preconditioning protected Gas from disuse atrophy preventing the up-regulation of atrogenes, we analyzed the expression of 17 key atrogenes involved in HU-induced muscle atrophy (Figure 3).

Following 1 day of HU, a significant up-regulation of mRNA expression of *FoxO3*, ubiquitin ligases atrogenes *MuRF1*, *Atrogin1*, *SMART* (Specific of Muscle Atrophy and Regulated by Transcription), *MUSA1* (Muscle Ubiquitin ligase of the SCF complex in Atrophy-1) and *FbxO31* (F-Box Protein 31), proteasome subunit atrogenes *Psmd11* (Proteasome 26S Subunit, Non-ATPase 11) and *Psme4* (Proteasome Activator Subunit 4), de-ubiquitinating enzyme atrogene *USP14* (Ubiquitin Specific Peptidase 14), ubiquitin atrogene *UBC* (Ubiquitin C), autophagy-related atrogenes *Cathepsin* l, *P62* (ubiquitin-binding protein p62) and *BNIP3* (BCL2 Interacting Protein 3), AMP deaminase atrogene (*Ampd3*), oxidative-stress-related atrogene *Mt1* (Metallothionein1), unfolded protein response atrogene *Gadd34* (Growth Arrest and DNA Damage-Inducible Protein), transcription regulators Smad2/3 atrogene (*TGIF,* TG-interacting factor) was found. Following 3 days of HU, *Atrogin1*, *FoxO3*, *FbxO31*, *Psmd11*, *Psme4*, *UBC*, *Cathepsin l*, *P62*, and *Gadd34*, were still significantly induced.

The expression of almost all atrogenes was lower 1 day after HU preceded by preconditioning (EX7 + HU1) than at HU1 with the only exception of *MUSA1*, *Mt1*, and *TGIF* whose reduced expression did not reach statistical significance (Figure 3). In preconditioned *mice*, following 3 days of HU, none of the atrogenes were less expressed compared to HU3. In the same *mice*, *Atrogin1*, *FbxO31*, *Psmd11*, *Psme4*, *UBC*, *Cathepsin l*, *P62*, and *Gadd34* were significantly induced compared to ground *mice*.

### 2.4. Akt/mTOR Pathway

**Figure 4:**
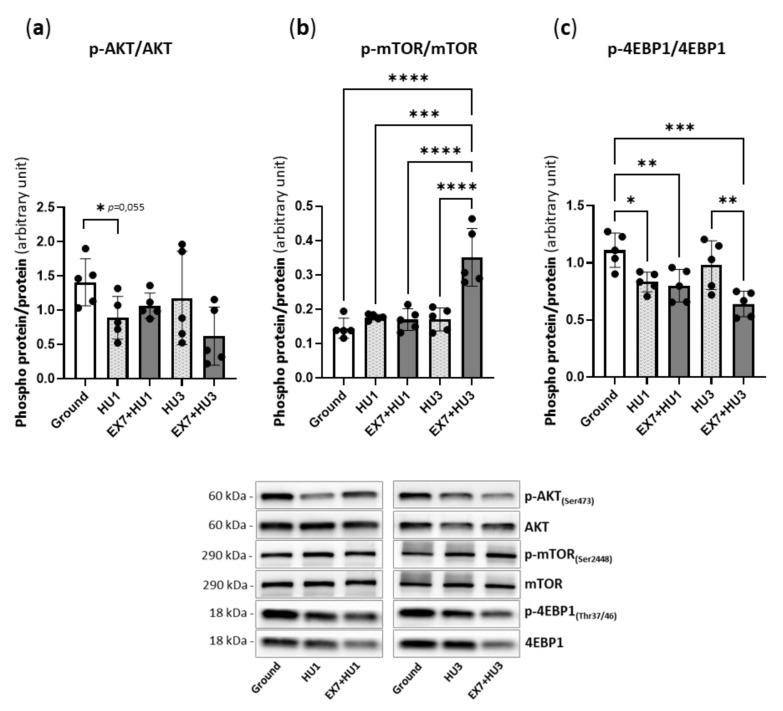
Physical preconditioning effect on phosphorylation of kinases of the Akt/mTOR pathway during disuse. Determination of activity levels of *p*-AKT (**a**), *p*-mTOR (**b**), and *p*-4EBP1 (**c**) by Western blot analysis of the ratio between the content in the phosphorylated (*p*) and total forms and relative representative Western blot. Ground: Control *mice*; HU1: *Mice* subjected to 1 day of hindlimb unloading; EX7 + HU1: *Mice* subjected to 7 days of physical preconditioning followed by 1 day of hindlimb unloading; HU3: *Mice* subjected to 3 days of hindlimb unloading; EX7 + HU3: *Mice* subjected to 7 days of physical preconditioning followed by 3 days of hindlimb unloading. Data are presented as means ± SD * *p* < 0.05 ** *p* < 0.005 *** *p* < 0.0005 **** *p* < 0.0001.

Figure 4 shows the adaptations of the AKT-mTOR pathway, which is known to contribute to the regulation of protein synthesis in skeletal muscle. Except for the decrease in the phosphorylation level of AKT (just over the limits of statistical significance, *p* = 0.055) and 4EBP1 at HU1, no significant changes in the phosphorylation levels of AKT, mTOR, and 4EBP1 were found during disuse. A significant increase in mTOR, together with a decrease in the 4EBP1 phosphorylation level, was found in EX7 + HU3 *mice*.

### 2.5. Markers of Mitochondrial Dynamics

Mitochondrial dynamics, i.e., the balance between mitochondrial fusion and fission, is a major determinant of mitochondrial function. Mitochondrial dysfunction can contribute to atrogenes’ activation causing an energy imbalance and AMPK activation.

**Figure 5:**
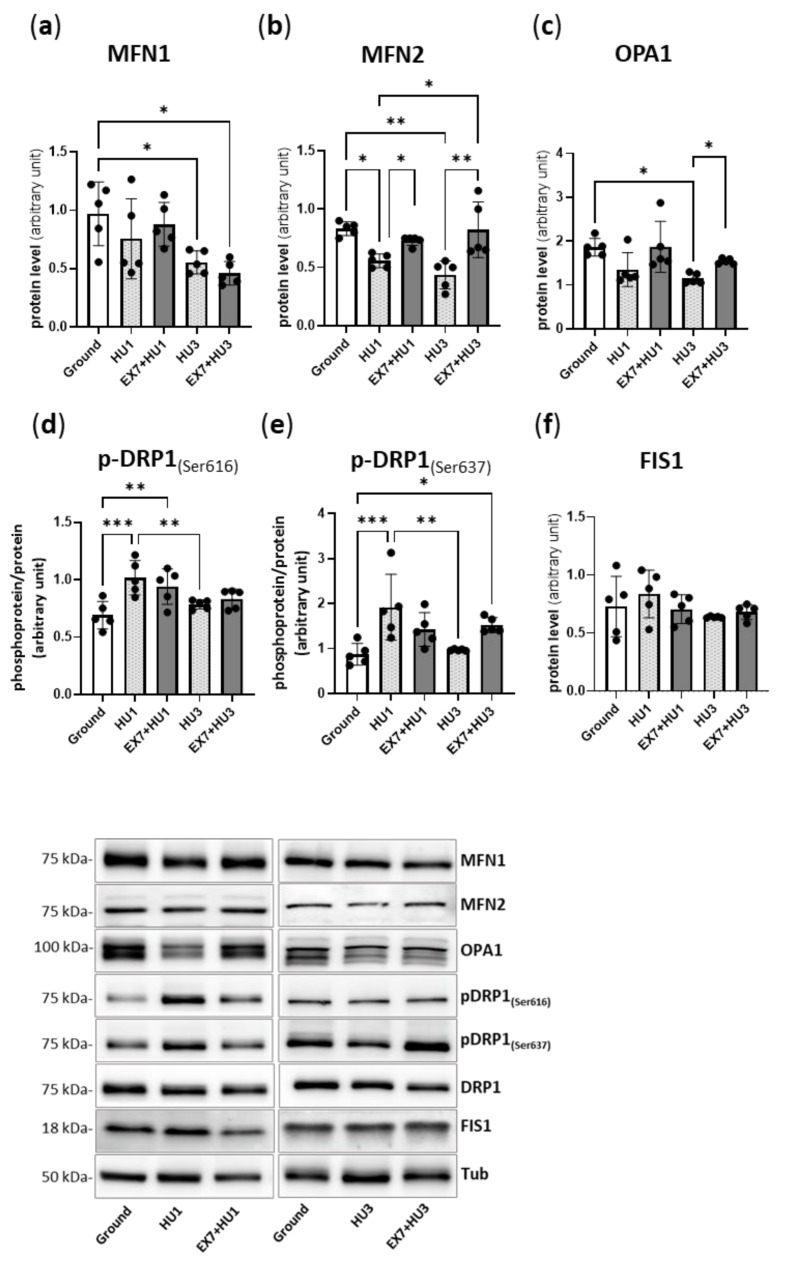
Physical preconditioning counteracts the decrease in MFN2 pro-fusion protein induced by disuse. Quantification of protein content of mitochondria dynamics markers by Western blotting. Pro-fusion proteins MFN1 (**a**), MFN2 (**b**), OPA1 (**c**), pro-fission proteins *p*-DRP1_(Ser616)_ (**d**), *p*-DRP1_(Ser637)_ (**e**), and FIS1 (**f**), and relative representative Western blot. Ground: Control *mice*; HU1: *Mice* subjected to 1 day of hindlimb unloading; EX7 + HU1: *Mice* subjected to 7 days of physical preconditioning followed by 1 day of hindlimb unloading; HU3: *Mice* subjected to 3 days of hindlimb unloading; EX7 + HU3: *Mice* subjected to 7 days of physical preconditioning followed by 3 days of hindlimb unloading. Protein levels were normalized for tubulin expression. The phosphorylation status of DRP1_(Ser616)_ and DRP1_(Ser637)_ was determined through the ratio between the content in the phosphorylated and total forms. Data are presented as means ± SD * *p* < 0.05 ** *p* < 0.005 *** *p* < 0.0005.

One day of disuse (HU1) resulted in a significantly lower content of the pro-fusion protein MFN2 with respect to the ground *mice* (Figure 5b). Such a reduction was also found at HU3 along with a significant reduction of MFN1 and OPA1 (Figure 5a,c).

Exercise preconditioning significantly counteracted MFN2 reduction both in EX + HU1 and EX7 + HU3 *mice*, as well as the OPA1 reduction in EX7 + HU3 *mice*.

The phosphorylation level of DRP1 at Ser-616 and at Ser-637 was found to be significantly higher at 1 day of disuse (HU1) and was not affected by physical preconditioning (Figure 5d,e). The protein level of pro-fission marker FIS1 was unchanged in all experimental groups (Figure 5f).

### 2.6. AMPK, PGC1α, and SIRT1

We investigated how the atrophy-related genes’ expression correlates with the known modulators of FoxO activity, AMPK, PGC1 α, and SIRT1, during disuse with and without exercise preconditioning.

Compared to ground *mice*, the phosphorylated form of AMPK was lower at HU1 and higher at HU3. Interestingly, preconditioning prevented AMPK activation, i.e., increased phosphorylation, at HU3.

**Figure 6:**
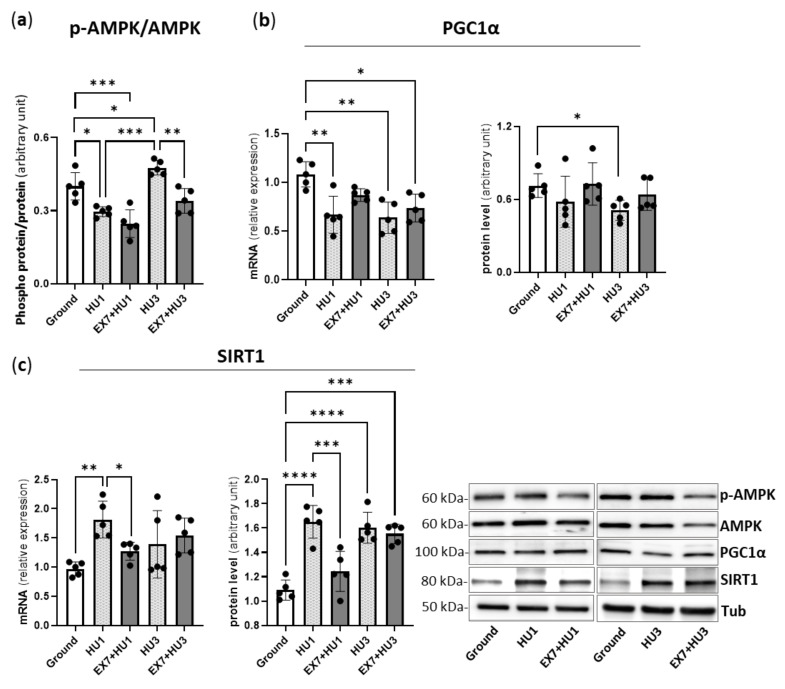
Effects of hindlimb unloading and exercise preconditioning on FoxO regulators. Activation level of AMP-kinase (**a**), Gene expression and protein content analysis of PGC1α (**b**), gene expression and protein content analysis of SIRT1 (**c**), and relative representative Western blot. Ground: Control *mice*; HU1: *Mice* subjected to 1 day of hindlimb unloading; EX7 + HU1: *Mice* subjected to 7 days of physical preconditioning followed by 1 day of hindlimb unloading; HU3: *Mice* subjected to 3 days of hindlimb unloading; EX7 + HU3: *Mice* subjected to 7 days of physical preconditioning followed by 3 days of hindlimb unloading. Data are presented as means ± SD * *p* < 0.05 ** *p* < 0.005 *** *p* < 0.0005 **** *p* < 0.0001. Gene expression levels were determined by RT-PCR analysis and normalized on *GAPDH*. Protein content levels were determined by Western blot analysis and normalized for tubulin. The activation level of AMP-kinase was determined by Western blot analysis of the ratio between the content of the phosphorylated and total form.

*PGC1α* expression was significantly lower both in HU1 and HU3 Gas than in ground Gas. The PGC1α protein content was lower in HU3 Gas compared to ground Gas. No significant modulation of *PGC1α* expression and protein content was found in preconditioned *mice* EX7 + HU1 and EX7 + HU3 with respect to HU1 and HU3, respectively (Figure 6b). However, PGC1α protein content was not different from the control in Ex7 + HU3.

*SIRT1* expression was found significantly upregulated in HU1 Gas in comparison to ground Gas. The SIRT1 protein level was found significantly higher than in ground *mice* at both HU1 and HU3. In EX7 + HU1, its expression level was found significantly lower than in HU1 and similar to the ground Gas. SIRT1 protein content was found at the ground level in EX7 + HU1 *mice* and at the HU3 level in EX7 + HU3 *mice* (Figure 6c). It appears that preconditioning prevented SIRT1 up-regulation at HU1, but not at HU3.

## 3. Discussion

Disuse atrophy is a major clinical problem. Mitochondrial dysfunction was shown to play a major role in muscle wasting [8,9,11,12,13]. Indeed, PGC1α over-expression could prevent disuse-induced muscle atrophy [8,13]. Being able to upregulate PGC1α expression and modulate mitochondrial function, exercise is an attractive tool to prevent disuse muscle atrophy. Despite recent evidence suggesting the capacity of exercise preconditioning to prevent disuse atrophy, the underlying mechanisms are not well characterized yet. We studied the impact of 7 days of endurance exercise preconditioning on (i) a large set of FoxO-dependent atrogenes that we previously indicated as the core machinery of protein breakdown in HU-induced gastrocnemius atrophy [30], (ii) markers of mitochondrial dynamics since we previously indicated pro-fusion protein deficiency as the major cause of gastrocnemius HU atrophy [13], and (iii) potential modulators of FoxO activity.

Endurance training and PGC1α/AMPK axis. We first ensured that the endurance exercise protocol employed was able to induce higher PGC1α expression and protein content in the gastrocnemius muscle. A single bout of endurance exercise activated AMPK and enhanced PGC1α expression and content (Figure 1). Following one week of daily endurance exercise, PGC1α expression and content were clearly enhanced, indicating the activation of the AMPK-PGC1α axis, consistently with previous observations [31].

Muscle atrophy and atrogenes’ expression. It is well known that the gastrocnemius goes through early atrophy following disuse [18,30,32], notwithstanding its predominant (~87%) MHC-2B isoform content and very low (~2%) MHC-1 content [30,32]. Consistently, we found a 13% loss of CSA following 3 days of HU (Figure 2).

Preconditioning prevented atrophy until 7 days of HU (Figure 2). Such a finding is consistent with the blunting of disuse atrophy following a single bout of endurance exercise [19] and 2 weeks of concurrent exercise training [18]. As expected for endurance exercise, the exercise protocol did not induce muscle fibers’ hypertrophy (Figure 2). Therefore, atrophy prevention was not simply due to the longer duration required to induce atrophy starting from a larger mass, but to qualitative changes in muscle proteins and intracellular pathways. Muscle mass protection was temporary, fading away within 14 days (Figure 2).

To characterize the molecular mechanism underlying muscle mass protection, we studied the expression of 17 FoxO-dependent atrogenes. Such atrogenes were selected based on their upregulation in the gastrocnemius with HU-induced atrophy and their suppression in gastrocnemius of FoxO knockout *mice* protected from HU atrophy [26]. Most atrogenes are involved in catabolic intracellular pathways: Ubiquitin ligases (*MuRF1*, *Atrogin1*, *SMART*, *MUSA1*, *FbxO31*), proteasome subunit (*Psmd11*, *Psme4*), de-ubiquitinating enzyme (*USP14*), ubiquitin gene (*UBC*), autophagy-related genes (*Cathepsin l*, *LC3*, *P62*, *BNIP3*), AMP deaminase gene (*Ampd3*), oxidative-stress-related gene (*Mt1*), unfolded protein response (*Gadd34*), transcription regulators Smad2/3 (TGIF).

Interestingly, the upregulation of all atrogenes started 1 day after unloading (Figure 3). We previously showed a generalized induction of atrogenes following 3 days of disuse [26]. Here we have shown that their largest changes occur as early as 1 day into HU and precede muscle atrophy, which occurs 3 days after HU.

Most atrogenes were downregulated in preconditioned *mice* 1 day after HU. Atrogenes’ expression resumed as early as 3 days following HU in preconditioned *mice* (Figure 3). Interestingly, no activation of most atrogenes was present in preconditioned *mice* 7 and 14 days after HU (Supplementary Figure S2). Considering that up-regulated mRNAs elicit a somewhat delayed increase in protein content and that proteins have variable half-lives, the blunting of the peak of atrogenes’ expression at 1 day (Figure 3) could account for muscle atrophy protection until 7 days. The rapid muscle atrophy that occurred between 7 and 14 days in preconditioned *mice* could be accounted for by the resumption of atrogenes’ expression at 3 days, given the almost normal atrogenes’ expression between 7 and 14 days of HU in preconditioned *mice*. Atrogenes’ expression following HU was therefore biphasic and clearly preceded muscle mass loss by several days. Such a phenomenon is already known for *MuRF1* and *Atrogin1* in muscle atrophy induced by hindlimb unloading [33], denervation, and spinal cord isolation [34], and is here extended to all atrogenes involved in hindlimb unloaded atrophy [26]. Interestingly, the transient nature of atrogenes’ expression could explain the previously observed lack of effects of preconditioning on *MuRF1* and *Atrogin1* [20] analyzed at a single time point of disuse. Moreover, considering that endurance exercise per se did not have any impact on the expression of several key atrogenes (Supplementary Figure S1), whereas a trend of higher expression of atrogenes in HU1 compared to HU3 and in EX7-HU3 compared to HU3 seems to occur (Figure 3), preconditioning might postpone but not eliminate a major atrogenes’ peak normally occurring 1 day after HU.

It is noteworthy that not all atrogenes induced by disuse were significantly downregulated by preconditioning 1 day after HU. This was the case of *Atrogin1*, *MUSA1*, *Mt1*, and *TGIF*. To exclude that this was due to an increase in proteolysis aimed at maintaining homeostasis during exercise [35], we verified atrogenes’ expression in ground *mice* subjected either to a single bout or to the one-week endurance exercise training used for preconditioning. We ruled out that the exercise protocol we employed was responsible *per se* for the induction of atrogenes (Supplementary Figure S1). Therefore, one possible explanation could be that the repressive effect of preconditioning on such genes occurred before 24 h and faded away at 24 h. Alternatively, they could be under the control of other transcription factors, besides FoxO, that are not affected by physical preconditioning. Indeed, it has been shown that deletion of all FoxO family members completely protected muscle mass from hindlimb unloading atrophy and reduced, but not abolished, the expression of major atrogenes, such as *MuRF1* and *Atrogin1*, and *Mt1* expression as well [26].

Atrogenes expression and mitochondrial dynamics. In the search for mechanisms underlying exercise-induced blunting of atrogenes expression and muscle mass protection, we studied PGC1α expression and content, AMPK activation, and markers of mitochondrial dynamics. It was shown that lower expression of PGC1α and mitochondrial dysfunction are major players in disuse-induced muscle atrophy [9]. PGC1α down-regulation can disinhibit FoxOs [5] and mitochondrial dysfunction can cause FoxOs activation through energy imbalance and AMPK activation [8,13,30,36].

We show that MFN1, MFN2, and OPA1 mitochondrial pro-fusion proteins content was reduced in response to disuse. The phenomenon started at HU1 (MFN2 downregulation) and clearly showed at HU3 (MFN1 and 2 and OPA1 down-regulation) (Figure 5). Such altered mitochondrial dynamics following HU could be supported by the downregulation of PGC1α. We show lower PGC1α expression starting at HU1 and lower protein content at HU3 (Figure 6). Consistently with the development of mitochondrial dysfunction suggested by altered mitochondrial dynamics, the activation of AMPK, the main sensor of cellular energy status, was higher following 3 days of HU (Figure 6) indicating an energy imbalance within muscle fibers. Collectively, the latter observations confirm that the very early activation of a metabolic program is a major player causing gastrocnemius muscle atrophy during HU [13].

Preconditioning maintained pro-fusion proteins content at the ground control levels during HU. Interestingly, a similar pro-fusion shift with exercise was reported both in *humans* [37] and animals [38]. Correction of the fusion proteins deficit is very important since the failure of this process can have detrimental consequences on muscle mass [9]. Muscle-specific ablation of MFN1 and MFN2 induces muscle atrophy [10], while the overexpression of OPA1 provides protection from denervation-induced muscle loss [11].

As previously shown [13], our results suggest no change in the mitochondrial fission process during early disuse (Figure 5), in accordance with the unchanged FIS1 protein level and the simultaneous increased phosphorylation of DRP1 at Ser-616 and at Ser-637 that activates and weakens, respectively, the ability of DRP1 to induce mitochondrial fission [39,40]. Similarly, fission was not affected by preconditioning with a tendency to decrease in EX7 + HU3 as suggested by the increase in phosphorylation of DRP1 at Ser-637 (Figure 5).

The observation that the enhanced activation of AMPK was prevented by preconditioning (Figure 6) indicates the normalization of energy efficiency and ATP production [41], consistently with the reversal of PGC1α and of disuse-induced fusion deficit. Such concurrent normalization of mitochondrial dynamics and the energy balance and blunting of muscle atrophy at EX7 + HU3 is consistent with our hypothesis that exercise preconditioning could reverse the metabolic program of muscle atrophy.

However, such a reversal was not sufficient to counteract the activation of atrogenes’ expression 3 days after HU. We previously showed that the overexpression of PGC1α in transgenic *mice* fully prevents HU-induced atrogenes up-regulation and muscle atrophy in gastrocnemius [13]. However, in transgenic *mice*, the PGC1α level was approximately 4-fold higher than the wild-type level all along HU. Preconditioning induced a 25% increase in PGC1α content (Figure 1), which was only at the ground level 1 and 3 days after HU (Figure 6). It appears that a much higher level of PGC1α is needed to stably prevent the disuse-induced peak of atrogenes’ expression than that achieved with in vivo preconditioning.

Potential FoxO regulators. Normal mitochondrial dynamics in EX7-HU3 suggest that some other factor is likely involved in the resumption of atrogenes’ expression, ultimately causing the disappearance of the protective effect of preconditioning after 14 days of HU.

The activity of FoxO transcription factors can be regulated by several post-translational modifications, including phosphorylation and acetylation. Consequently, many different kinases and acetyltransferase or deacetylase positively or negatively modulate FoxO activity. Besides AMPK, these include SIRT1 deacetylase and AKT.

SIRT1 [28,29] is known to be involved in controlling protein degradative pathways, specifically via FoxO transcription factors. SIRT1 has been shown to activate both *FoxO3* gene expression and to deacetylate FoxO3, thereby activating FoxO DNA binding and elevating the expression of target genes [28]. We found a correlation between the deacetylase SIRT1 levels and the FoxO-mediated expression of the atrogenes’ program (Figure 6). Following 1 and 3 days of HU, SIRT1 expression and content and FoxO and all atrogenes’ expression were upregulated. SIRT1 expression and content and FoxO and atrogenes’ expression were blunted 1 day after HU preceded by preconditioning. SIRT1 expression and content up-regulation and atrogenes’ expression resumed in preconditioned *mice* 3 days after HU. The latter observations are consistent with FoxO activity in the skeletal muscle being negatively regulated by acetylation. However, data are controversial. Some reports indicate that acetylation of FoxO transcription factors causes decreased activity [42], and others show that deacetylation of FoxO cause either decreased activity [43,44,45] or increased activity [28,46].

A strong induction of SIRT1 expression and activity [47] as well as a decrease in SIRT1 [48] was found in the condition of denervation muscle atrophy. A decrease in SIRT1 was also observed with caloric restriction [45]. Importantly, acetylation reduces FoxO3 transcription capability and causes the cytosolic “relocalization” of FoxO3, lowering the activation of the atrophy program in conditions both of denervation [42] and immobilization [49]. It is, therefore, reasonable to speculate that deacetylation of FoxO mediated by SIRT1 promotes the nuclear localization of FoxO3 and the atrophic program. On the other hand, we cannot rule out that SIRT1 induction may serve as a compensatory protective mechanism against a massive increase in protein degradation during unloading.

FoxO transcription factors are downstream the PI3K/AKT/mTOR pathway that induces protein synthesis and promotes the inactivation of FoxO by inducing its phosphorylation and nuclear exclusion [50]. Here we found an early (1 day of HU) and transient decline in the AKT activation level (*p* = 0.055) (Figure 4) suggesting its role in dephosphorylation and nuclear translocation of FoxO and therefore in the activation of atrogenes very early after HU. The reduction of 4EBP1 further supports the early AKT pathway decrease. The transient decrease in the synthetic pathway might have a role in the activation of the catabolic system through the AKT-FoxO axis rather than a role in the process of mass loss, in agreement with its normalization observed after 3 days of disuse. The latter finding is consistent with previous observations showing no alterations in the phosphorylation states of the anabolic phosphatidyl-inositol 3-kinase (PI3K/AKT/mTOR) pathway in gastrocnemius, at least up to 3 days of disuse [13,26].

In summary, exercise preconditioning prevents early muscle atrophy, mitochondrial dysfunction, and energy imbalance, consistently with our hypothesis that exercise can counteract the disuse-induced alteration in mitochondrial dynamics. However, the normalization of AMPK activation and PGC1α is not sufficient to prevent the resumption of atrogenes’ expression as early as 3 days into HU and progressive muscle atrophy. Muscle mass protection is temporary and fades away between 7 and 14 days of HU. Protection might be prolonged using a longer or more intense exercise training.

## 4. Materials and Methods

Six-month-old male C57BL/6 *mice* (Charles River Laboratories, Wilmington, MA, USA) were used. *Mice* were randomized into the following groups: Control *mice* (Ground), *mice* subjected to 1 (HU1), 3 (HU3), 7 (HU7), and 14 (HU14) days of hindlimb unloading (HU), *mice* subjected to a single bout of exercise sacrificed immediately or after 3 h (Ground SB-EX + 0 h, Ground SB-EX + 3 h), *mice* subjected to 7 days of endurance exercise followed by 1 day of suspension (EX7 + HU1), 3 days of suspension (EX7 + HU3), 7 days of suspension (EX7 + HU7), and 14 days of suspension (EX7 + HU14), and *mice* subjected to 7 days of endurance exercise sacrificed 3 h after the last section of exercise (Ground EX7). Six animals per group were used. As previously described [8,13], animals subjected to HU were suspended individually in special cages by a thin string tied at one end to the tail and at the other end to the top of the cage; the length of the string was adjusted to allow the animals to move freely on their forelimbs, while the body was inclined at 30–40 degrees from the horizontal plane. All *mice* had access to water and food ad libitum.

### 4.1. Exercise Training

*Mice* were acclimatized to the motorized treadmill (Exer 3/6 Treadmill, Columbus Instruments, Columbus OU, USA) by running 30 min/day for 3 days at 5 m/min, 0° inclination. Exercise was performed as follows: 5 min of warmup at 5 m/min after which time the speed was increased 1 m/min every minute until the speed of 10 m/min. Then the *mice* ran 20 min at 10 m/min, 20 min at 11 m/min, and finally, 20 min at 12 m/min [51]. *Mice* subjected to physical preconditioning before HU ran for seven consecutive days. Animals of all experimental groups were fasted 2 h prior to the sacrifice. *Mice* were sacrificed by cervical dislocation, and gastrocnemius (Gas) muscles were dissected and immediately frozen in liquid nitrogen and stored at −80 °C.

### 4.2. Cross-Sectional Area (CSA) Analysis

Muscle fibers’ CSA was determined in the mid-belly region of Gas muscles as previously described [30]. Briefly, muscle serial transverse sections (10 μm thick) were obtained from Gas and stained with hematoxylin-eosin. Images of the stained sections were captured from a light microscope (Leica DMLS, Wetzlar, Germany) equipped with a camera (Leica DFC 280, Wetzlar, Germany). Fibers’ CSA was measured with Image J analysis software (NIH, Bethesda, MD, USA) and expressed in square micrometers. At least 5000 fibers per sample were measured.

### 4.3. Western Blot Analysis

Frozen muscle samples were pulverized and immediately re-suspended in a lysis buffer (20 mM Tris-HCl, 1% Triton ×100, 10% glycerol, 150 mM NaCl, 5 mM EDTA, 100 mM NaF, and 2 mM NaPPi supplemented with 1× protease, phosphatase inhibitors (Sigma-Aldrich, St Louis, MO, USA), and 1mM PMSF). The homogenate obtained was kept in ice for 20 min and then centrifuged at 18,000× *g* for 20 min at 4 °C. The supernatant was stored at −80 °C until ready to use. Protein concentrations were evaluated for each sample and equal amounts of muscle samples were loaded on gradient precast gels purchased from Bio-Rad (AnyKd; Hercules, CA, USA). After the gel run, proteins were electro-transferred to PVDF membranes at 35 mA overnight. The membranes were incubated in 5% Milk for 2 h, rinsed with TBST buffer (0.02 M Tris and 0.05 M NaCl, pH 7.4–7.6), and subsequently probed with specific primary antibodies (see below). Thereafter, the membranes were incubated in an HRP-conjugated secondary antibody. The protein bands were visualized by an enhanced chemiluminescence method in which luminol was excited by peroxidase in the presence of H_2_O_2_ (ECL Select, GE Healthcare, Litle Chalfont, UK). The content of each protein investigated was assessed by determining the brightness–area product of the protein band normalized to tubulin content.

### 4.4. Antibodies

Antibodies used were anti-mouse Tubuline (1:3000 Sigma Aldrich, St Louis, MO, USA); anti-rabbit PGC-1α (1: 1000, Abcam, Cambridge, UK); anti-rabbit *p*-AMPK_(thr 172)_ (1:1000 Cell Signaling, Danvers, MA, USA); anti-rabbit AMPK (1:1000 Cell Signaling, Danvers, MA, USA); anti-mouse MFN1 (1:1000 Abcam, Cambridge, UK); anti-rabbit MFN2 (1:1000 Abcam, Cambridge, UK); anti-rabbit OPA1 (1:3000 Abcam, Cambridge, UK); anti-rabbit *p*-DRP1_(ser616)_ (1:1000 Cell Signalling, Danvers, MA, USA); anti-rabbit *p*-DRP1_(ser637)_ (1:1000 Cell Signalling, Danvers, MA, USA); anti-rabbit DRP1 (1:3000 Cell Signalling, Danvers, MA, USA); anti-rabbit FIS1 (1:1000 Abcam, Cambridge, UK); anti-rabbit *p*-mTOR_(Ser2448)_ (1:1000 Cell Signalling, Danvers, MA, USA); anti-rabbit mTOR (1:1000 Cell Signalling, Danvers, MA, USA); anti-rabbit *p*-AKT_(Ser473)_ (1:2000 Cell Signalling, Danvers, MA, USA); anti-rabbit AKT (1:2000 Cell Signalling, Danvers, MA, USA); anti-rabbit *p*-4EBP1_(Thr37/46)_ (1:1000 Cell Signalling, Danvers, MA, USA); anti-rabbit 4EBP1 (1:1000 Cell Signalling, Danvers, MA, USA); anti-mouse SIRT1 (1:1000 Cell Signalling, Danvers, MA, USA); anti-mouse IgG (1:5000 Dako North America Inc., Carpinteria, CA, USA); anti-rabbit IgG (1:10,000 Cell Signaling, Danvers, MA, USA).

### 4.5. Gene Expression Analysis 

Total RNA was extracted from Gas muscles using the SV Total RNA isolation kit (Promega, Madison, WI, USA). The RNA concentration was measured using a Nano Drop instrument (ThermoScientific, Waltham, MA, USA) and 400 ng was used to generate cDNA with SuperScript III reverse transcriptase (Invitrogen, Carlsbad, CA, USA). The cDNA was analyzed by quantitative RT-PCR (Applied Biosystems AB7500) using an SYBR Green PCR kit (Applied Biosystems, Foster City, CA, USA) or GoTaq^®^ Probe qPCR Master Mix (Qiagen, Hilden, Germany) and the data were normalized to GAPDH content. Oligonucleotide primers were provided by Sigma Aldrich (St. Louis, MO, USA) and listed in Table 1. Differentially expressed genes were determined using a default threshold of 0.6. The difference between Ct (cycle threshold) values was calculated for each mRNA by taking the mean Ct of duplicate reactions and subtracting the mean Ct of duplicate reactions for the reference RNA measured on an aliquot from the same RT reaction (ΔCt = Ct target gene—Ct reference gene). All samples were then normalized to the ΔCt value of a calibrator sample to obtain a ΔΔCt value (ΔCt target—ΔCt calibrator) (comparative method).

### 4.6. Statistical Analysis

Data were expressed as means ± SD. The statistical significance of differences between two means was evaluated by Student’s *t*-test. The statistical significance of differences between more than two means was evaluated by one-way ANOVA followed by the Newman–Keuls multiple-comparisons test. The two-way ANOVA followed by the Bonferroni post hoc test was used for comparisons between groups of preconditioned and unloaded samples at different durations of HU. The level of significance was set at *p* < 0.05. The statistical analysis was evaluated using GraphPad Prism software (GraphPad Software, San Diego, CA, USA).

## Figures and Tables

**Table 1:** Oligonucleotide primers.

Gene	Forward Primer (5′–3′)	Reverse Primer (5′–3′)
*Ampd3*	GCGGAGAAGGTGTTTGCTA	CAGTCTTGTTGTGTTGGCATC
*Atrogin1*	GCAAACACTGCCACATTCTCTC	CTTGAGGGGAAAGTGAGACG
*BNIP3*	TTCCACTAGCACCTTCTGATGA	GAACACCGCATTTACAGAACAA
*Cathepsin l*	GTGGACTGTTCTCACGCTCAAG	TCCGTCCTTCGCTTCATAGG
*FbxO31*	GTATGGCGTTTGTGAGAACC	AGCCCCAAAATGTGTCTGTA
*FoxO3*	ACCTTCGTCTCTGAACTCCTTG	CTGTGGCTGAGTCAGTCTGAAG
*Gadd34*	AGAGAAGACCAAGGGACGTG	CAGCAAGGAATGGACTGTG
*GAPDH*	CACCATCTTCCAGGAGCGAG	CCTTCTCCATGGTGGTGAAGAC
*Mt1*	GCCTGCAAGAACTGCAAGTG	CCTTTGCAGACACAGCCCT
*MUSA1*	TCGTGGAATGGTAATCTTGC	CCTCCCGTTTCTCTATCACG
*MuRF1*	ACCTGCTGGTGGAAAACATC	ACCTGCTGGTGGAAAACATC
*p62*	CCCAGTGTCTTGGCATTCTT	AGGGAAAGCAGAGGAAGCTC
*PGC1α*	ACCCCAGAGTCACCAAATGA	CGAAGCCTTGAAAGGGTTATC
*Psme4*	AGGACGAGCAGAAGAACCTG	AATAGTTAGAGCCTGTGGTGGAG
*SMART*	TCAATAACCTCAAGGCGTTC	GTTTTGCACACAAGCTCCA
*Psmd11*	GAGTTCCAGAGAGCCCAGTC	AACCCAGTTCAAGGATGCTC
*Usp14*	CACGAGTTGCTTCGTATTCC	TTCAGGGTTCCTCCTTTCAC
*UBC*	CGTCGAGCCCAGTGTTACCACC	ACCTCCCCCATCACACCCAAGA
*Tgif*	TTTCCTCATCAGCAGCCTCT	CTTTGCCATCCTTTCTCAGC

## Data Availability

The raw data are available upon request.

## Supplementary Materials

The following are available online at https://www.mdpi.com/article/10.3390/ijms23010148/s1.

## References

[1] Mazzucco S., Agostini F., Biolo G. (2010). Inactivity-mediated insulin resistance is associated with upregulated pro-inflammatory fatty acids in *human* cell membranes. Clin. Nutr..

[2] Booth F.W., Roberts C.K., Laye M.J. (2012). Lack of exercise is a major cause of chronic diseases. Compr. Physiol..

[3] Bonaldo P., Sandri M. (2013). Cellular and molecular mechanisms of muscle atrophy. Dis. Model. Mech..

[4] Pedersen B.K., Saltin B. (2015). Exercise as medicine-evidence for prescribing exercise as therapy in 26 different chronic diseases. Scand. J. Med. Sci. Sports.

[5] Sandri M., Lin J., Handschin C., Yang W., Arany Z.P., Lecker S.H., Goldberg A.L., Spiegelman B.M. (2006). PGC-1alpha protects skeletal muscle from atrophy by suppressing FoxO3 action and atrophy-specific gene transcription. Proc. Natl. Acad. Sci. USA.

[6] Wenz T., Rossi S.G., Rotundo R.L., Spiegelman B.M., Moraes C.T. (2009). Increased muscle PGC-1alpha expression protects from sarcopenia and metabolic disease during aging. Proc. Natl. Acad. Sci. USA.

[7] Geng T., Li P., Yin X., Yan Z. (2011). PGC-1α promotes nitric oxide antioxidant defenses and inhibits FOXO signaling against cardiac cachexia in *mice*. Am. J. Pathol..

[8] Cannavino J., Brocca L., Sandri M., Bottinelli R., Pellegrino M.A. (2014). PGC1-alpha over-expression prevents metabolic alterations and soleus muscle atrophy in hindlimb unloaded *mice*. J. Physiol..

[9] Romanello V., Sandri M. (2015). Mitochondrial Quality Control and Muscle Mass Maintenance. Front. Physiol..

[10] Chen H., Vermulst M., Wang Y.E., Chomyn A., Prolla T.A., McCaffery J.M., Chan D.C. (2010). Mitochondrial fusion is required for mtDNA stability in skeletal muscle and tolerance of mtDNA mutations. Cell.

[11] Varanita T., Soriano M.E., Romanello V., Zaglia T., Quintana-Cabrera R., Semenzato M., Menabò R., Costa V., Civiletto G., Pesce P. (2015). The OPA1-dependent mitochondrial cristae remodeling pathway controls atrophic, apoptotic, and ischemic tissue damage. Cell Metab..

[12] Favaro G., Romanello V., Varanita T., Andrea Desbats M., Morbidoni V., Tezze C., Albiero M., Canato M., Gherardi G., De Stefani D. (2019). DRP1-mediated mitochondrial shape controls calcium homeostasis and muscle mass. Nat. Commun..

[13] Cannavino J., Brocca L., Sandri M., Grassi B., Bottinelli R., Pellegrino M.A. (2015). The role of alterations in mitochondrial dynamics and PGC-1alpha over-expression in fast muscle atrophy following hindlimb unloading. J. Physiol..

[14] Safdar A., Abadi A., Akhtar M., Hettinga B.P., Tarnopolsky M.A. (2009). miRNA in the regulation of skeletal muscle adaptation to acute endurance exercise in C57Bl/6J male *mice*. PLoS ONE.

[15] Norrbom J., Sundberg C.J., Ameln H., Kraus W.E., Jansson E., Gustafsson T. (2004). PGC-1alpha mRNA expression is influenced by metabolic perturbation in exercising *human* skeletal muscle. J. Appl. Physiol..

[16] Russell A.P., Feilchenfeldt J., Schreiber S., Praz M., Crettenand A., Gobelet C., Meier C.A., Bell D.R., Kralli A., Giacobino J.P. (2003). Endurance training in *humans* leads to fiber type-specific increases in levels of peroxisome proliferator-activated receptor-gamma coactivator-1 and peroxisome proliferator-activated receptor-alpha in skeletal muscle. Diabetes.

[17] Powers S.K., Bomkamp M., Ozdemir M., Hyatt H. (2020). Mechanisms of exercise-induced preconditioning in skeletal muscles. Redox. Biol..

[18] Theilen N.T., Jeremic N., Weber G.J., Tyagi S.C. (2018). Exercise preconditioning diminishes skeletal muscle atrophy after hindlimb suspension in *mice*. J. Appl. Physiol.

[19] Fujino H., Ishihara A., Murakami S., Yasuhara T., Kondo H., Mohri S., Takeda I., Roy R.R. (2009). Protective effects of exercise preconditioning on hindlimb unloading-induced atrophy of *rat* soleus muscle. Acta Physiol..

[20] Yoshihara T., Tsuzuki T., Chang S.W., Kakigi R., Sugiura T., Naito H. (2019). Exercise preconditioning attenuates hind limb unloading-induced gastrocnemius muscle atrophy possibly via the HDAC4/Gadd45 axis in old *rats*. Exp. Gerontol..

[21] Sandri M. (2008). Signaling in muscle atrophy and hypertrophy. Physiology.

[22] Glover E.I., Yasuda N., Tarnopolsky M.A., Abadi A., Phillips S.M. (2010). Little change in markers of protein breakdown and oxidative stress in *humans* in immobilization-induced skeletal muscle atrophy. Appl. Physiol. Nutr. Metab..

[23] Bodine S.C., Latres E., Baumhueter S., Lai V.K., Nunez L., Clarke B.A., Poueymirou W.T., Panaro F.J., Na E., Dharmarajan K. (2001). Identification of ubiquitin ligases required for skeletal muscle atrophy. Science.

[24] Sandri M. (2016). Protein breakdown in cancer cachexia. Semin. Cell Dev. Biol..

[25] Milan G., Romanello V., Pescatore F., Armani A., Paik J.H., Frasson L., Seydel A., Zhao J., Abraham R., Goldberg A.L. (2015). Regulation of autophagy and the ubiquitin-proteasome system by the FoxO transcriptional network during muscle atrophy. Nat. Commun..

[26] Brocca L., Toniolo L., Reggiani C., Bottinelli R., Sandri M., Pellegrino M.A. (2017). FoxO-dependent atrogenes vary among catabolic conditions and play a key role in muscle atrophy induced by hindlimb suspension. J. Physiol..

[27] Romanello V., Guadagnin E., Gomes L., Roder I., Sandri C., Petersen Y., Milan G., Masiero E., Del Piccolo P., Foretz M. (2010). Mitochondrial fission and remodelling contributes to muscle atrophy. EMBO J..

[28] Brunet A., Sweeney L.B., Sturgill J.F., Chua K.F., Greer P.L., Lin Y., Tran H., Ross S.E., Mostoslavsky R., Cohen H.Y. (2004). Stress-dependent regulation of FOXO transcription factors by the SIRT1 deacetylase. Science.

[29] An B.S., Tavera-Mendoza L.E., Dimitrov V., Wang X., Calderon M.R., Wang H.J., White J.H. (2010). Stimulation of Sirt1-regulated FoxO protein function by the ligand-bound vitamin D receptor. Mol. Cell Biol..

[30] Brocca L., Pellegrino M.A., Desaphy J.F., Pierno S., Camerino D.C., Bottinelli R. (2010). Is oxidative stress a cause or consequence of disuse muscle atrophy in *mice*? A proteomic approach in hindlimb-unloaded *mice*. Exp. Physiol..

[31] Egan B., Carson B.P., Garcia-Roves P.M., Chibalin A.V., Sarsfield F.M., Barron N., McCaffrey N., Moyna N.M., Zierath J.R., O’Gorman D.J. (2010). Exercise intensity-dependent regulation of peroxisome proliferator-activated receptor. coactivator-1 alpha mRNA abundance is associated with differential activation of upstream signalling kinases in *human* skeletal muscle. J. Physiol..

[32] Desaphy J.F., Pierno S., Liantonio A., Giannuzzi V., Digennaro C., Dinardo M.M., Camerino G.M., Ricciuti P., Brocca L., Pellegrino M.A. (2010). Antioxidant treatment of hindlimb-unloaded mouse counteracts fiber type transition but not atrophy of disused muscles. Pharm. Res..

[33] Hanson A.M., Harrison B.C., Young M.H., Stodieck L.S., Ferguson V.L. (2013). Longitudinal characterization of functional, morphologic, and biochemical adaptations in *mouse* skeletal muscle with hindlimb suspension. Muscle Nerve.

[34] Sacheck J.M., Hyatt J.P., Raffaello A., Jagoe R.T., Roy R.R., Edgerton V.R., Lecker S.H., Goldberg A.L. (2007). Rapid disuse and denervation atrophy involve transcriptional changes similar to those of muscle wasting during systemic diseases. FASEB J. Off. Publ. Fed. Am. Soc. Exp. Biol..

[35] Bell R.A., Al-Khalaf M., Megeney L.A. (2016). The beneficial role of proteolysis in skeletal muscle growth and stress adaptation. Skelet Muscle.

[36] Herzig S., Shaw R.J. (2018). AMPK: Guardian of metabolism and mitochondrial homeostasis. Nat. Rev. Mol. Cell Biol..

[37] Axelrod C.L., Fealy C.E., Mulya A., Kirwan J.P. (2019). Exercise training remodels *human* skeletal muscle mitochondrial fission and fusion machinery towards a pro-elongation phenotype. Acta Physiol..

[38] Iqbal S., Ostojic O., Singh K., Joseph A.M., Hood D.A. (2013). Expression of mitochondrial fission and fusion regulatory proteins in skeletal muscle during chronic use and disuse. Muscle Nerve.

[39] Chang C.R., Blackstone C. (2007). Cyclic AMP-dependent protein kinase phosphorylation of Drp1 regulates its GTPase activity and mitochondrial morphology. J. Biol. Chem..

[40] Hu C., Huang Y., Li L. (2017). Drp1-Dependent Mitochondrial Fission Plays Critical Roles in Physiological and Pathological Progresses in Mammals. Int. J. Mol. Sci..

[41] Sergi D., Naumovski N., Heilbronn L.K., Abeywardena M., O’Callaghan N., Lionetti L., Luscombe-Marsh N. (2019). Mitochondrial (Dys)function and Insulin Resistance: From Pathophysiological Molecular Mechanisms to the Impact of Diet. Front. Physiol..

[42] Bertaggia E., Coletto L., Sandri M. (2012). Posttranslational modifications control FoxO3 activity during denervation. Am. J. Physiol. Cell Physiol..

[43] Motta M.C., Divecha N., Lemieux M., Kamel C., Chen D., Gu W., Bultsma Y., McBurney M., Guarente L. (2004). Mammalian SIRT1 represses forkhead transcription factors. Cell.

[44] Yang Y., Hou H., Haller E.M., Nicosia S.V., Bai W. (2005). Suppression of FOXO1 activity by FHL2 through SIRT1-mediated deacetylation. EMBO J..

[45] Lee D., Goldberg A.L. (2013). SIRT1 protein, by blocking the activities of transcription factors FoxO1 and FoxO3, inhibits muscle atrophy and promotes muscle growth. J. Biol. Chem..

[46] Beharry A.W., Sandesara P.B., Roberts B.M., Ferreira L.F., Senf S.M., Judge A.R. (2014). HDAC1 activates FoxO and is both sufficient and required for skeletal muscle atrophy. J. Cell Sci..

[47] Chabi B., Adhihetty P.J., O’Leary M.F., Menzies K.J., Hood D.A. (2009). Relationship between Sirt1 expression and mitochondrial proteins during conditions of chronic muscle use and disuse. J. Appl. Physiol..

[48] Chacon-Cabrera A., Gea J., Barreiro E. (2017). Short- and Long-Term Hindlimb Immobilization and Reloading: Profile of Epigenetic Events in Gastrocnemius. J. Cell Physiol..

[49] Senf S.M., Sandesara P.B., Reed S.A., Judge A.R. (2011). p300 Acetyltransferase activity differentially regulates the localization and activity of the FOXO homologues in skeletal muscle. Am. J. Physiol. Cell Physiol..

[50] Sartori R., Romanello V., Sandri M. (2021). Mechanisms of muscle atrophy and hypertrophy: Implications in health and disease. Nat. Commun..

[51] Shefer G., Rauner G., Stuelsatz P., Benayahu D., Yablonka-Reuveni Z. (2013). Moderate-intensity treadmill running promotes expansion of the satellite cell pool in young and old *mice*. FEBS J..

