# Supplementary figures and images for: Exercise Preconditioning Blunts Early Atrogenes Expression and Atrophy in Gastrocnemius Muscle of Hindlimb Unloaded Mice
Source: Int J Mol Sci. 2021 Dec 23;23(1):148. doi: 10.3390/ijms23010148 (PMC8745338; doi:10.3390/ijms23010148)

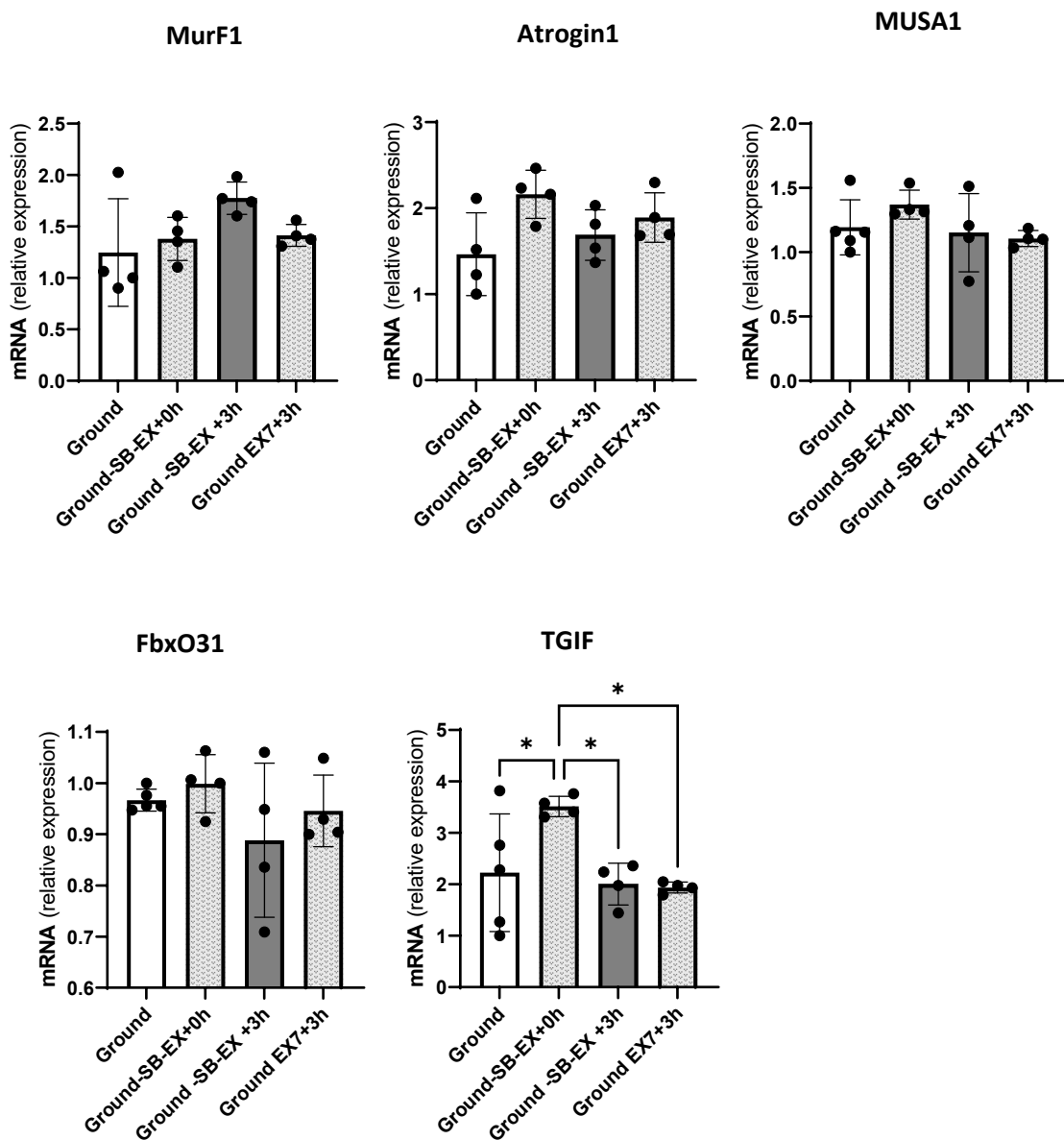

Figure S1

Supplement: Supplementary file 1 [file ijms-23-00148-s001.zip › Figure S1.pdf]

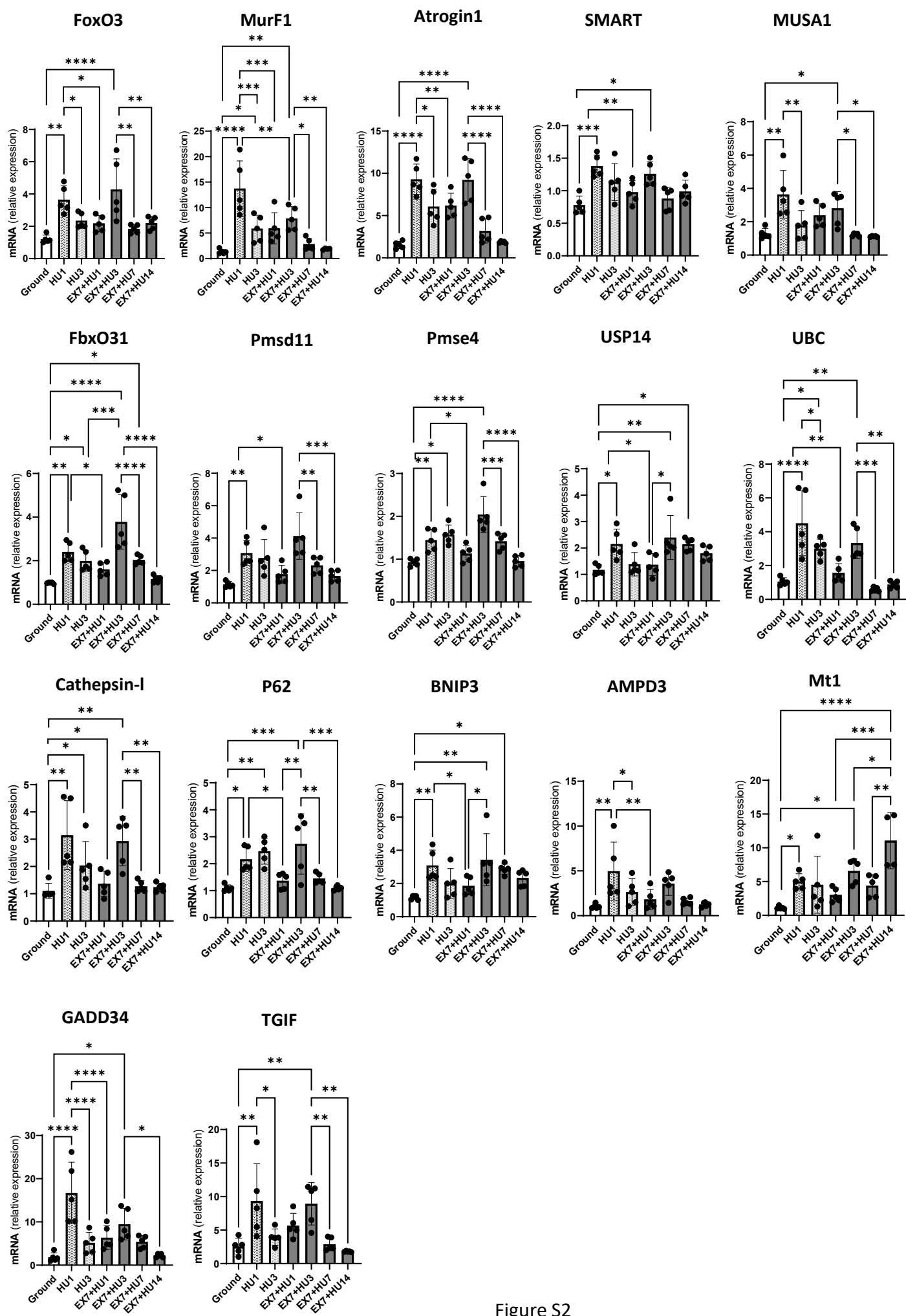

Figure S2

Supplement: Supplementary file 1 [file ijms-23-00148-s001.zip › Figure S2.pdf]
